# Biosensors Based on Inorganic Composite Fluorescent Hydrogels

**DOI:** 10.3390/nano13111748

**Published:** 2023-05-26

**Authors:** Pavel Sokolov, Pavel Samokhvalov, Alyona Sukhanova, Igor Nabiev

**Affiliations:** 1 Life Improvement by Future Technologies (LIFT) Center, Skolkovo, 143025 Moscow, Russia; socolovpm87@mail.ru (P.S.); p.samokhvalov@gmail.com (P.S.); 2 Laboratory of Nano-Bioengineering, National Research Nuclear University MEPhI (Moscow Engineering Physics Institute), 115522 Moscow, Russia; 3 Laboratoire de Recherche en Nanosciences, LRN-EA4682, Université de Reims Champagne-Ardenne, 51100 Reims, France; alyona.sukhanova@univ-reims.fr

**Keywords:** inorganic fluorescent hydrogel, fluorescent nanocrystal, biosensor

## Abstract

Fluorescent hydrogels are promising candidate materials for portable biosensors to be used in point-of-care diagnosis because (1) they have a greater capacity for binding organic molecules than immunochromatographic test systems, determined by the immobilization of affinity labels within the three-dimensional hydrogel structure; (2) fluorescent detection is more sensitive than the colorimetric detection of gold nanoparticles or stained latex microparticles; (3) the properties of the gel matrix can be finely tuned for better compatibility and detection of different analytes; and (4) hydrogel biosensors can be made to be reusable and suitable for studying dynamic processes in real time. Water-soluble fluorescent nanocrystals are widely used for in vitro and in vivo biological imaging due to their unique optical properties, and hydrogels based on these allow the preservation of these properties in bulk composite macrostructures. Here we review the techniques for obtaining analyte-sensitive fluorescent hydrogels based on nanocrystals, the main methods used for detecting the fluorescent signal changes, and the approaches to the formation of inorganic fluorescent hydrogels via sol–gel phase transition using surface ligands of the nanocrystals.

## 1. Introduction

The current approach to engineering sensor diagnostic platforms is aimed at obtaining autonomous and versatile biosensors not requiring expensive equipment for analysis or time-consuming sample preparation, with the result available within minutes. This concept is referred to as point-of-care (PoC) or bedside diagnostics, but it is not restricted to medicine. Such sensors would be also useful for food testing, e.g., in rapid bacterial contamination tests, and for environment testing, e.g., in portable air or water contamination sensors. The PoC devices include: lateral flow tests and enzyme-linked immunosorbent assay (ELISA), which are used for identifying antibodies and antigens in bacterial or viral infection diagnoses [1]; detecting markers of different diseases [2,3]; testing food samples for antibiotic contamination [4]; performing blood coagulation screening [5], etc.; portable plasmonic platforms based on surface plasmon resonance for detecting the markers of diseases, including bacterial, viral, and autoimmune ones [6,7]; methods for the detection of viral, bacterial, and parasitic infections by means of the polymerase chain reaction and derivatives of this method [8,9]; the so-called bench-top PoC testing analyzers used for spectrophotometric measurement of substrate amount and enzyme activity; hematological particle counting; immunoassay; sensor-based blood-gas analysis; hemostaseological coagulation analysis [10,11], etc. Many current miniature sensors were originally developed similarly to the so-called labs-on-a-chip sensors used in more complex systems. Since fluids are the natural environment of most biological molecules, microfluidic flow cells and microfluidic chips are among the most common formats of these devices for the analysis of biological molecules [12]. Advanced microfluidic devices can combine many detection technologies, such as antibody–antigen interaction and different varieties of the polymerase chain reaction. Macroscale analogs of these miniature devices include liquid chromatographs, flow cytometers, suspension chip systems, and classical enzyme immunoassay kits.

Miniaturization offers many advantages, including a high throughput ensured by parallel assays, short time of analysis, low manufacturing costs, and small volumes of both the sample and the reagents, which is important in the case of expensive or hazardous reagents [13,14,15]. However, miniaturization also entails some limitations compared to traditional methods. For example, ELISA uses a larger sample volume and a greater number of detection molecules, which ensures a more intense absolute signal than microfluidic-based sensors [16]. Another example of the case when larger is better is chromatographic separation or purification of the sample, whose efficiency is directly proportional to the physical size of the column [17]. Thus, miniaturization alone is not sufficient for the successful use of microfluidic detection systems; it is also necessary to search for the ways of enhancing the sensitivity by increasing the effective interaction volume between the detecting molecules and the analyte and using more sensitive tags for the detection. Fluorescence detection is the most sensitive of all optical detection methods [18,19], provided that the optimal type of the fluorescence label, e.g., organic dyes, fluorescent proteins, or fluorescent nanocrystals (NCs), has been selected for each specific task. The problem of increasing the effective interaction volume in microsystems is particularly urgent, because miniaturization reduces the overall number of detecting and reporter molecules [20]. While, e.g., in chromatography, branched-surface resins functionalized with selective molecules are used to increase the effective interaction volume, this is not always applicable in microfluidic systems, because opaque media impede fluorescence detection [21]. In addition, the particles of such a medium can move with the fluid flow, which complicates detection and may lead to blocking of microfluidic channels or media washout from the microfluidic chip, thus compromising its analytical properties.

One of the most promising trends in the development of microfluidic sensors is the use of fluorescent labels based on inorganic NCs incorporated in a three-dimensional (3D) optically transparent polymeric matrix permeable for fluid flow. There are two most common types of such matrices: (1) matrices based on organic polymers with embedded fluorescent NCs [22,23], and (2) composite hydrogels formed from colloidal NCs via sol–gel phase transition [24]. These hydrogels have a porous branched structure, which is suitable for immobilization of sensory, affinity, and signal elements, and they are hydrophilic, which is necessary for the unhindered passage of the analytes [25]. The analyte can be specifically recognized by the structure of the gel material itself [26]; otherwise, the functionalization of the hydrogel with affinity labels can be used for this purpose. The signal function can also be fulfilled by the hydrogel itself in some cases; e.g., there are gel materials that lose their structure when the pH is changed, either by dissolution or swelling [27]. However, the most common way is to incorporate signal molecules that generate fluorescent [28] or chromogenic [29] signals, or change their electrochemical [30] or conductive [31] properties.

In the next section we will discuss in detail the approaches to obtaining NC-based inorganic composite fluorescent hydrogels, as well as the principles of optical signal generation in hydrogel-based biosensors that rely on fluorescence detection.

## 2. Approaches to Obtaining Fluorescent Gels

Fluorescent hydrogels constitute one of the main components of biosensors. In addition to the optical signal generation, they are responsible for the immobilization of affinity molecules and the maintenance of the 3D structure of the biosensor. In general, fluorescent gels are obtained by incorporating fluorescent labels, such as organic dyes [32], proteins/peptides [33], aromatic hydrocarbons [34], metal nanoparticles [35], or NCs [36], into the gel structure. The main requirements for fluorescent labels are the capability for multiplexed detection, high detection sensitivity and fluorescent signal intensity, photostability, and a reliable and convenient procedure for immobilizing fluorescent labels in the hydrogel. Multiplexed detection requires multiple labels with different fluorescence spectra but coinciding or overlapping absorption spectra, so that they can be excited simultaneously with a single source of radiation of a given wavelength. There are many different organic dyes and proteins that fluoresce in various spectral regions from visible to near-infrared. However, they have wider fluorescence spectra and narrower absorption spectra than fluorescent NCs, which makes it difficult to simultaneously excite and detect the fluorescent signal [37]. Fluorescent NCs exhibit the quantum size effect, allowing fluorescence spectra to be tailored to specific applications by selecting the NC size [38]. The NC photostability, i.e., the retention of their fluorescence properties upon exposure to light, is higher than that of organic fluorescent dyes [39], which makes it possible to increase the sensitivity through long-term signal accumulation, as well as to perform prolonged detection, e.g., in studying prolonged processes or sequentially analyzing several samples, without a decline in the signal intensity with time.

**Figure 1:**
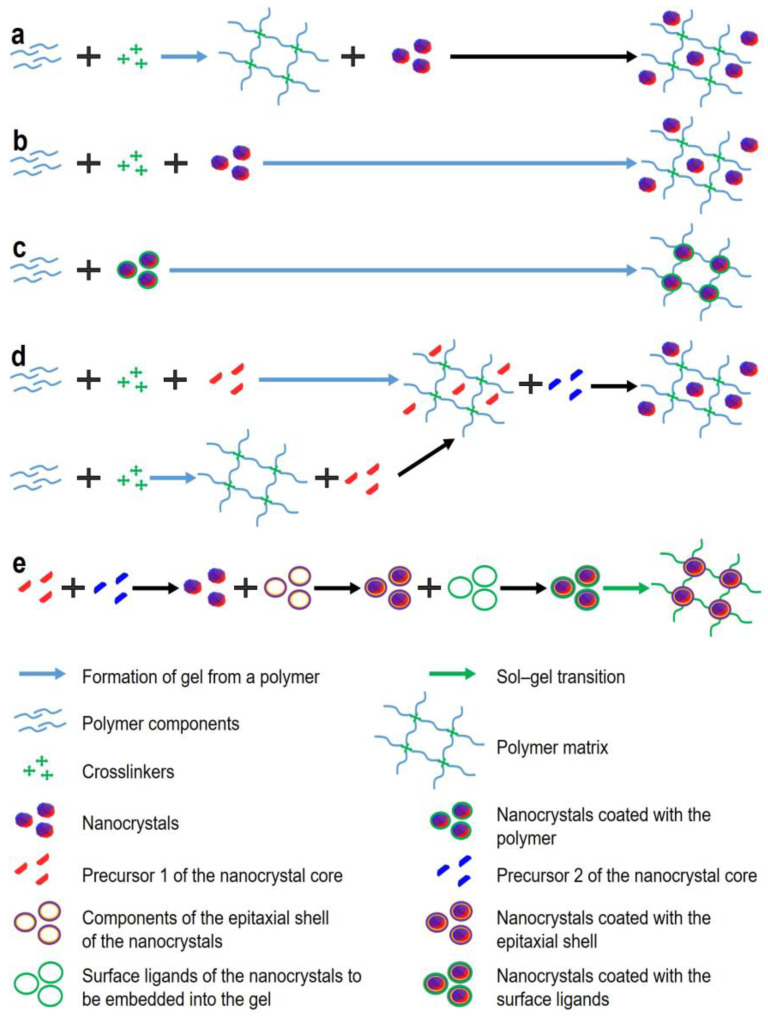
Methods for obtaining fluorescent gels. (**a**) post-synthesis loading of gel with NC; (**b**) loading of gel with NC during gel formation; (**c**) direct incorporation of NC into gel; (**d**) synthesis of NC during gel formation; (**e**) sol-gel approach of fluorescent gel formation.

Fluorescent gels can be obtained using two fundamentally different approaches. The first approach is to embed fluorescent labels into the polymer structure, which is suitable for almost all types of fluorescent labels, both organic and inorganic. The second approach is based on the phenomenon of sol–gel phase transition and is traditionally used with inorganic fluorescent NCs (Figure 1). Below we will focus on the methods of inclusion of NC labels, because, as noted above, they have several advantages over other fluorescent labels and are the most promising for the use in this type of biosensors. In the first method of incorporation of fluorescent labels into a gel, the polymer and crosslinker components are mixed and left to polymerize. When the gel has been formed, it is incubated together with the fluorescent labels so that they diffuse into the resulting matrix (Figure 1a). Li et al. used this method to incorporate carbon dots into polymethacrylic acid gels [40]. Carbon dots were immobilized in the gel due to the formation of hydrogen bonds between the amino groups of the dot shell and the carboxyl groups of the polymer. However, this method does not provide immobilization of the fluorescent labels as robust as that provided by the second method, because further manipulations or pumping of the samples in polar solvents may cause disruption of hydrogen bonds and washout of the fluorescent labels from the gel. In the second method, the components for gel formation and fluorescent labels are mixed simultaneously, so that the labels become enclosed in the pores of the gel during its formation (Figure 1b). It should be noted that both methods require additional procedures to ensure uniform distribution of the labels within the gel, such as sonication [41]. The third method, based on crosslinking of hydrogel monomers and NC surface chemical agents (Figure 1c), yields hydrogels containing uniformly distributed fluorescent NCs without additional treatment of the gel. Because the density of such gels directly depends on the NC content, this method is sometimes less suitable for obtaining gels with the desired pore size and fluorescence intensity. For example, Xu et al. functionalized the ZnS NC surface with chemical agents that induced polymerization of methyl methacrylate or glycidyl methacrylate and acted as crosslinkers for the monomers [42]. Yang et al. described the formation of fluorescent gels based on acrylic acid and acrylamide containing CdS NCs with the use of gamma radiation as a polymerization initiator [43]. Coordination interaction between the Cd atoms on the NC surface and the N atoms of the polyacrylamide gel resulted in a homogeneous NC distribution in the gel and the absence of NC aggregation even if their concentration was increased. In addition, the polymer molecules can interact with heavy metal ions of not only the NCs, but also their precursors (Figure 1d). Jiang et al. powdered dried cellulose hydrogel and placed it into a solution of heavy metal precursors: cadmium chloride pentahydrate and sodium sulfide [44]. Positively charged Cd^2+^ ions were electrostatically and chemically absorbed on the gel surface, and their interaction with S^2−^ ions gave rise to the growth of NCs steadily embedded in the gel structure. However, this method is disadvantageous compared to the above two examples, in that it allows gel encapsulation of only core NCs lacking an epitaxial inorganic shell, and the latter is required to improve the stability and optical properties of NCs. In addition, this method imposes limitations on the NC synthesis procedure; e.g., it does not allow precise control of the NC size and, hence, their fluorescence spectrum. Figure 1e shows the method of obtaining fluorescent hydrogels via a sol–gel phase transition, when NC surface ligands are involved in gel formation. Gaponic et al. [45] have reported an example of the formation of such a gel formed of CdTe NCs whose surface is functionalized with compounds containing thiol groups. It is worth noting that this method of obtaining composite inorganic fluorescent hydrogels is the most versatile, because it allows using previously synthesized and characterized fluorescent core/shell NCs, ensures their uniform distribution in the hydrogel, and completely preserves the optical properties of the NCs by preventing their aggregation. Before reviewing the methods for modification of the NC surface and induction of gel formation, we will touch upon the principles of detection of target analytes in biosensors based on fluorescent gels.

## 3. Mechanisms of the Formation of Stable Gels for Biosensing Applications

Fluorescent NCs are semiconductor structures several nanometers, or sometimes several tens of nanometers, in size. Their optical properties, such as fluorescence and absorption spectra, depend on the NC size, structure, and composition. NC cores most commonly consist of elements of groups III–V, II–VI, or groups IV–VI of the periodic system. The core may be coated with an outer epitaxial inorganic shell, which improves the NC optical properties and makes them more resistant to environmental factors [46]. The epitaxial shell made of a wide-gap material (ZnS or CdS) reduces the nonradiative recombination of charge carrier pairs on the surface defects of the core and improves fluorescence quantum yield. The NC composition determines not only their optical properties, but also their toxicity. CdSe, CdTe (elements of groups II–VI), PbS, and PbSe (elements of groups IV–VI) NCs are highly toxic materials because of the heavy metals, which not only makes these NCs hazardous to synthesize and use, but also causes problems with their disposal. InP (elements of groups III–V) NCs do not contain heavy metals, but their optical properties strongly depend on their size and structure [47]. Ternary NCs with cores containing three elements of groups I–III–VI, e.g., CuInS_2_ (CIS) and AgInS_2_ (AIS) NCs, constitute a better alternative. In order to increase fluorescence quantum yield, epitaxial ZnS or ZnS_X_/Se_1−X_ shells are applied onto the CIS and AIS cores, which reduces charge recombination on the core surface [48].

The modification of the NC shell surface with ligands is necessary for rendering them hydrophilic and more biocompatible and for functionalizing them with various organic molecules, as well as for forming hydrogels through the sol–gel phase transition. Colloidal stability of NCs is typically provided by repulsive forces between NCs due to ligands bound to their surfaces after synthesis or post-synthetic ligand exchange. Gelation can involve either weakening of these stabilizing forces or creation of attraction forces between NCs. In some cases, gels can form spontaneously through aggregation of colloidally unstable nanoparticles [49], but only the formation of gels from stable colloidal NC dispersions allows assembling fluorescent NC gels with desired properties. Destabilization via ligand removal weakens the short-range interactions that hold the NCs apart and induce aggregation due to van der Waals attraction. However, this can lead to the formation of aggregates in the gel and loss of fluorescence properties of the NCs [50,51]. The sol–gel transition is usually initiated by optical irradiation, thermal treatment, or chemical destabilization of the sol, either spontaneous or caused by chemical agents. Gaponik et al. [45] found that storage of aqueous colloidal solutions of CdTe NCs coated with thioglycolic and mercaptopropionic acids during the synthesis eventually leads to spontaneous transition into a gel capable of maintaining its shape and integrity when flushed with buffer solutions. In this case, the gel was spontaneously formed due to oxidation of the stabilizing agents or partial hydrolysis of the NC ligands. This resulted in the formation of a structure several millimeters in size, and the NCs contained in it retained the optical properties characteristic of the NC solution. In addition, some parts of the gel had a red or yellow–green color. This indicated hierarchical organization of individual clusters, which were formed at different rates from NCs of different sizes. In practice, however, spontaneous gel formation due to aging is rarely used, because it does not allow obtaining reproducible results.

**Figure 2:**
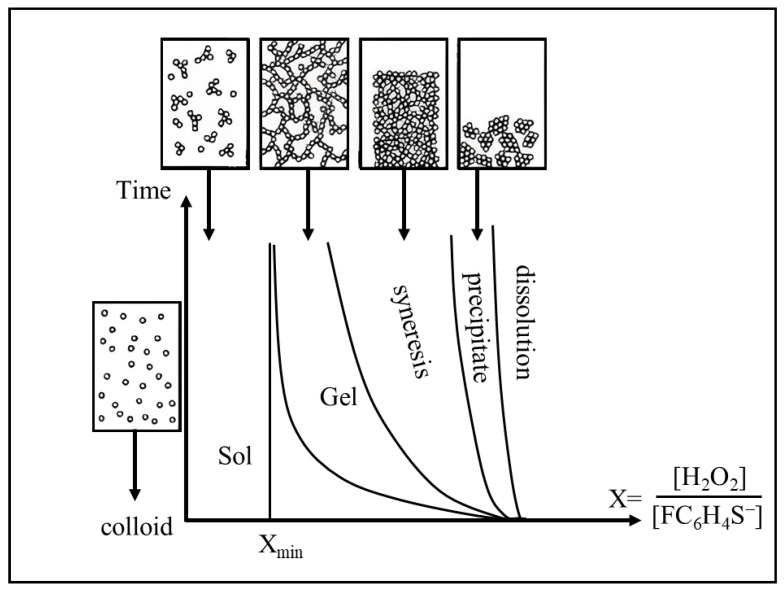
Schematic representation of the time evolution of CdS colloids after oxidation by aqueous hydrogen peroxide solutions depending on the X, molar ratio between H_2_O_2_ and the grafted thiolate in the colloid. Reprinted with permission from Gacoin et al. [57]. Copyright 2023 American Chemical Society.

The formation of NC hydrogels induced by chemical factors that destabilize colloidal solutions was described, e.g., by Brock et al. [52]. In that case, CdSe/ZnS NCs coated with mercaptoundecanoic acid formed hydrogels upon addition of the oxidizing agent tetranitromethane (TNM). The authors noted that the optical properties of the core/shell NCs were better preserved compared with core NCs without a shell. It should also be noted that a strong oxidizing agent, such as TNM or hydrogen peroxide, can degrade the surface of core NCs, thereby decreasing the quality of the resultant fluorescent gels. Metal ions are an example of less aggressive agents that destabilize colloidal solutions. Hewa-Rehinduwage et al. [53] described the effects of Ni^2+^, Co^2+^, Ag^+^, and Zn^2+^ ions on the gel formation from CdS and CdSe NCs functionalized with thioglycolic acid and mercaptoundecanoic acid. The method proposed by these authors is also suitable for the formation of fluorescent gels on the surface of electrodes, which is relevant for the development of biosensors of various types. Gel formation can also be induced by adding an antisolvent or changing the dielectric properties of the buffer solution [54]. Lesnyak et al. [55] described the gel formation from NCs coated with 5-mercaptomethyltetrazole, with cadmium acetate as the gelation initiator. The gelation process itself took from several seconds to two or three weeks, depending on the amount of cadmium acetate added. This method yielded reproducible results, the synthesized gels remained transparent, and their constituent NCs retained their optical properties, as evidenced by the small, if any, difference between the positions of the absorption maxima of the colloidal NC solution and the gel. At the same time, the fluorescence maximum was shifted by about 15 nm towards longer wavelengths, which was probably due to the energy transfer from smaller NCs to larger ones. This hypothesis was also supported by the shorter fluorescence decay in colloidal NCs than in gel-like ones, in which additional channels for nonradiative energy transfer were formed. These authors also tested the reversibility of chemically induced gelation. For this purpose, ethylenediaminetetraacetic acid (EDTA), which effectively chelates metal ions, was added to the resulting hydrogel. When EDTA was added in an amount equimolar to cadmium acetate, which was used to initiate gelation, the gel structure was destroyed, the solution became transparent, the NC fluorescence intensity was restored by 80%, and the fluorescence spectrum returned to its original shape. Subsequent increase in the cadmium acetate concentration again led to gel formation. Thiol-containing ligands are not the only ones used to form hydrogels. For example, Yao et al. showed the formation of gels from colloidal solutions of CdTe NCs coated with 16-mercaptohexadecanoic acid (MHA) or trioctylphosphine oxide (TOPO) ligands [56]. In both cases, gelation was chemically induced by the addition of TNM, with gelation of the MHA-coated NCs starting after 3–4 h, and that of the TOPO-coated NCs, after 3–4 days. The gelation of MHA-coated NCs could also be induced using a photochemical method, e.g., by irradiating the NCs with a mercury lamp for 9 h. Gacoin et al. [57] used H_2_O_2_ to obtain gels from CdS NCs functionalized with 4-fluorophenylthiol (4FPT) ligands via chemical oxidation of 4FPT. Figure 2 shows the time evolution of CdS NC colloids after oxidation by H_2_O_2_ as dependent on the ratio (X) between the added H_2_O_2_ and the amount of the 4FPT ligands on the NC surface. The addition of a small amount of H_2_O_2_ did not induce any noticeable change in the solution until the threshold value (X_min_) was reached, after which gelation was observed. At X ≥ X_min_, oxidized thiolate caused the gelation of the sol after some time as a consequence of the shrinkage of the solid network related to the solvent expulsion from the pores. In the case of very high amounts of the oxidant added (typically, 5X_min_), gelation was not observed, and the particles only precipitated. At even higher concentrations of the oxidant, the particles were partly dissolved as a result of the oxidation of the sulfide ions of CdS.

Fluorescent gels can also be obtained by using cross-coupling molecules that bind with the surface of the NCs. This provides additional control over the assembly of the gel structure. Yan et al. [58] proposed a hyperbranched macromolecule that could serve not only as an excellent ligand to stabilize CdSe/ZnS and CdSe NCs, but also as a good gelator inducing the formation of a reversible and multiresponsive fluorescent gel. Coupling molecules can act as bridges in the formation of NC gels, but non-adsorbing molecules can have a similar effect due to depletion interactions. Saez Cabezas et al. [59] reported a gelation method based on physical interactions between NCs, namely, short-range depletion attraction balanced by long-range electrostatic repulsion. The latter occurred upon removal of the native organic ligands that passivate tin-doped indium oxide NCs, while the former was induced by mixing with small polyethylene glycol chains. An increase in the polyethylene glycol concentration resulted in two gelation windows, the first arising from bridging effects and the second being attributed to depletion attraction according to phase behavior predicted by a unified theoretical model.

**Figure 3:**
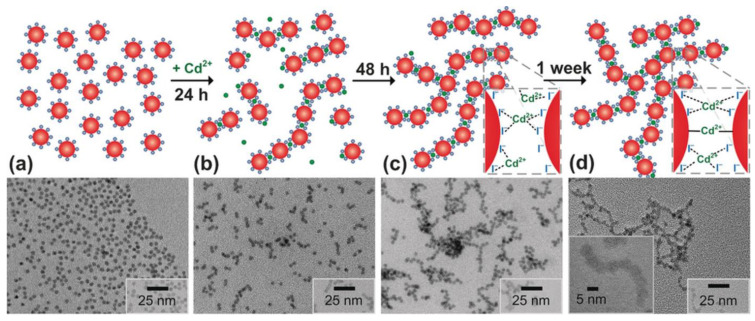
Schematic representation of the gelation process and TEM images showing the gelation of CdSe(I^−^) NCs (**a**) 24 h (**b**), 48 h (**c**), and 1 week (**d**) after the addition of Cd^2+^ linker. Dotted lines, covalent bond; red circle, NC; green circle, Cd^2+^; blue circle, I^−^. Reprinted with permission from Eychmüller et al. [60]. Copyright 2016 John Wiley and Sons.

In addition, NC gels can be obtained by adding linkers that form bonds between NC surface ligands. This ensures a controlled gelation process and allows new functional and sensor groups to be introduced into fluorescent gels. Sayevich et al. [60] demonstrated the assembly of all-inorganic colloidal NCs into gels mediated by NC linking with appropriate ions. They synthesized CdSe NCs with diameters of about 4.5 nm and replaced the surface ligands with NH_4_I, which resulted in I^−^-capped NCs. The gelation was induced by adding cadmium acetate in N-methylformamide. The time-course of gelation is schematically shown in Figure 3. The authors assumed that the aging process included the formation of strong covalent bonds, including I–Cd–I and Se–Cd–Se, accompanied by interfusion of the NCs, which determined the rigid framework of the gel structure. The lack of reversibility in the case of using strong complexation agents (EDTA) additionally confirmed the formation of strong bonds between the NCs. Transmission electron microscopy (TEM) showed the aggregation of CdSe(I^−^) NCs at early stages of gelation (Figure 3). First, after 24 h, chains were formed; later up to 48 h, they branched to give rise to more complex fractal aggregates, which acted as building blocks for the construction of a well-connected gel network after 1 week.

Another method of initiating gelation is the irradiation of a colloidal NC solution with light. It should be noted that, in this method, it is important to control the radiation power and wavelength range, so as to avoid NC damage because of the photoinduced etching of the surface [61]. High-power radiation causes degradation of NCs and the formation of a dispersed powdery precipitate. Irradiation in the infrared range also impairs the optical properties of the gels because of excessive heating. On the other hand, short-wavelength radiation is effectively absorbed by surface organic ligands, which interferes with uniform gel formation. The photoinduced gelation method can be used to form gels from NCs whose surface is functionalized with thiol-containing ligands, such as thioglycolic or mercaptopropionic acid [45]. When irradiated with a 150 W xenon lamp, the first signs of gelation appear within 5–10 h, and centrifugation of the solution for several hours after the start of gelation at low rates stimulates the gelation and allows the gel to be purified from the solvent. Note that the fluorescence intensity only slightly decreases during gelation. This decrease may be caused by aggregation of NCs due to the disruption of the outer organic shell. Minor changes in the position of the fluorescence peak may be related to changes in the size of NCs due to their degradation or, conversely, growth in response to irradiation.

Hewa-Rehinduwage et al. [62] studied the reversible electrochemical gelation of CdS, ZnS, and CdSe NCs. In the case of the CdS NCs, gelation occurs via electrochemical oxidation of surface-bound thiolate ligands in the form of dithiolates and solvation of Cd ions, after which the surface chalcogenides are oxidized and dichalcogenide bonds are formed between the NCs. The redox nature of the dichalcogenide bonds allows the gel network to be disassembled by reducing the NC surface to obtain chalcogenides at negative potentials. ZnS and CdSe NC hydrogels are formed in a similar way. The choice of ligands and specific modes of gel formation also depend on the parameters of the initial colloidal solution, such as the NC concentration and size [63,64]. The ligands required for gelation can either be added to NCs at the stage of synthesis [55] or, if this is impossible, replaced post-synthetically via surface ligand exchange [65].

## 4. Biosensors Based on Fluorescent Gels

**Figure 4:**
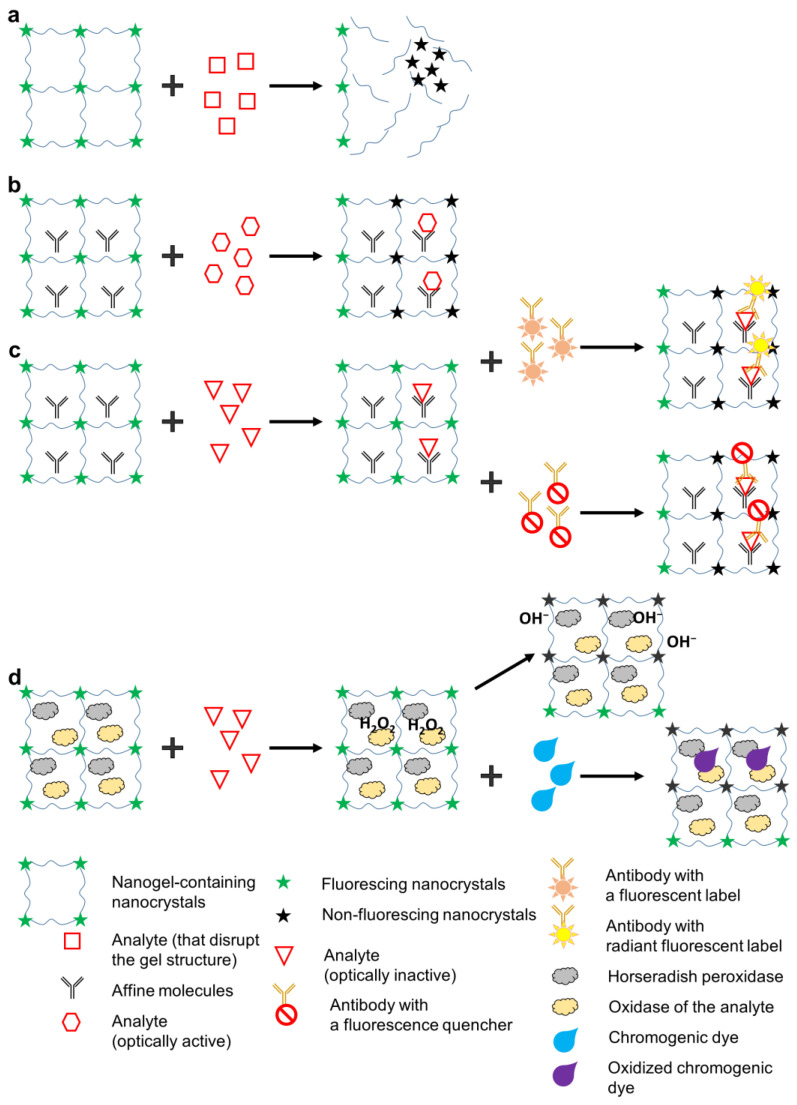
Variants of detection of target analyte molecules in fluorescent gels: (**a**) the target analyte disrupts the gel structure; (**b**) the analyte is optically active; (**c**) the analyte is optically inactive; (**d**) enzyme-dependent fluorescence quenching.

Biosensing using fluorescent gels employs various approaches to analyte detection, but all of them involve the recording of changes in the fluorescent properties of the gel. Depending on the analyte detected and the design of the biosensor, the changes in the optical properties may be of two types: first, quenching of NC fluorescence due to energy transfer (e.g., via the Förster resonance energy transfer, FRET), structural degradation of the fluorescent NCs, or their aggregation due to gel structure disruption; or second, changes in the fluorescence spectrum due to the activation of new fluorescent labels. The specific mechanism of the change in the optical properties often depends not only on the properties of the fluorescent NCs and the gel, but also on the characteristics of the analyte and additional sensory molecules contained in the biosensor. In some cases, specific binding of the analyte is ensured by NC functionalization with surface ligands, such as cyclodextrins [66] or (2-hydroxyethyl) dithiocarbamate [67]. However, this is applicable almost exclusively to relatively simple analytes, such as metal ions [68], polyaromatic molecules [69], and NO^2−^ [70]. Specific recognition of more complex organic molecules, such as antibodies or other proteins, requires the use of affinity molecules, e.g., antibodies. The main principles of detection used in fluorescent hydrogel biosensors are shown in Figure 4. One of the simplest detection principles (Figure 4a) is used with analytes that disrupt the gel structure upon contact, which results in NC aggregation and decreased fluorescence intensity. This detection principle was used by Bhattacharya et al. [71] for the detection of bacteria. They made a fluorescent gel from 6-O-(O-O′-dilauroyltharyl)-D-glucose and carbon NCs and, after its polymerization, added cultures of *Bacillus* and *Staphylococcus* strains. During the growth of the culture, the bacterial cells secreted esterases catalyzing the ester cleavage reaction. As a result, the fluorescence signal decreased, depending on the number of added bacteria, due to the degradation of the gel matrix and aggregation of the NCs. Two main mechanisms have been assumed for explaining the red shift of the NC emission spectra and fluorescence quenching upon aggregation: electronic coupling and exciton energy transfer [72]. This approach is well suited for the cases where the target analyte can degrade the gel matrix; however, it entails irreversible destruction of the biosensor, which makes biosensors of this type hardly practicable. The detection principle shown in Figure 4b is applicable to analytes that either specifically quench NC fluorescence via various excitation relaxation mechanisms or fluoresce themselves (i.e., they are optically active). For example, metal ions can accept electrons to be reduced, thereby decreasing the NC fluorescence intensity [73]. Many organic compounds can act as electron acceptors in the Förster resonance energy transfer, which also decreases the NC fluorescence intensity or leads to the formation of an additional fluorescence peak [74]. This principle is often used for the detection of metal ions, the selectivity being ensured by functionalization of the NC surface with a ligand that selectively interacts with a specific ion, e.g., Fe^3+^ [75], Cr^6+^ [76], or Cu^2+^ [77]. Yuan et al. used lignin and celluolose nanofiber hydrogels containing carbon dots (CDs) for the detection of hexavalent chromium [78]. In this biosensor, fluorescence was quenched due to electrostatic attraction between Cr^6+^ ions and the CD surface. Ehtesabi et al. [79] described a tetracycline biosensor based on a hydrogel with embedded fluorescent CDs. The hydrogel was composed of alginate crosslinked with divalent calcium ions. The CDs were then captured into the alginate network, where calcium ions interacted with the carboxyl groups on their surface. Tetracycline bound to the CD carboxyl groups and facilitated the nonradiative recombination of excitons through effective electron transfer, leading to fluorescence quenching. Ruiz-Palomero et al. developed fluorescent nanocellulose hydrogels based on sulfur- and nitrogen-codoped graphene quantum dots for sensing laccase [80]. Laccase weakly interacted with the graphitic layers stabilized by nanocellulose, which led to energy transfer and, hence, quenching of the fluorescence of graphene quantum dots immersed in the hydrogel. The advantages of this detection principle are that it does not require additional optically active reporter labels and can be used for simultaneous detection of several analytes if they fluoresce in different regions of the optical spectrum. Its disadvantage is a low versatility, because most analytes are optically inactive. In addition, its selectivity is low: fluorescence may be quenched by optically active compounds other than the target analyte that the sample may contain. Figure 4c shows the scheme of the detection of optically inactive analytes. In this case, auxiliary optically active labels are used that specifically bind the analyte and either quench the optical signal or fluoresce in a different spectral region. Antibodies or oligonucleotides can be used as capture molecules [81], and organic dyes [82], plasmonic nanoparticles [83], graphene [84], or other compounds causing changes in the fluorescence of hydrogels are used as optically active labels. This mechanism is similar to that of the detection of optically active samples. Chen et al. [85] developed a hydrogel-based biosensor for quantative detection of progesterone. The biosensor was composed of a polyhistidine-tagged transcription factor linked to CdSe/CdS/ZnS NCs and a fluorophore-modified cognate DNA embedded in a poly(ethylene glycol)-based hydrogel. Upon exposure to progesterone, DNA dissociated from the NC–transcription factor–DNA assembly, which stopped FRET from the NC to the fluorophore and, hence, quenched the fluorescence. The disadvantage of this detection principle is the need for an additional optical component. On the other hand, this significantly increases the specificity of detection, because the analyte to be detected should be additionally recognized by a specific optically active label. In the fourth detection method (Figure 4d), two enzymes, oxidase and horseradish peroxidase (HRP), are introduced into the pores of a fluorescent gel. The former enzyme specifically oxidizes the analyte to form hydrogen peroxide, and the latter one uses hydrogen peroxide as a cofactor to catalyze the formation of OH^−^ radicals, which weaken the hydrogel fluorescence [86], and the oxidation of the chromogenic dye, which shifts the spectral maximum of the absorption signal, also contributing to the quenching of the gel fluorescence [87]. For example, Cho et al. [86] developed a glucose biosensor operating on this principle. They immobilized fluorescent carbon NCs, rhodamine 6G, glucose oxidase, and HRP in a hydrogel. Upon excitation at a wavelength of 360 nm, the blue fluorescence of the carbon NCs was quenched by the bienzymatic reaction with glucose and the formation of OH^−^ radicals, while the fluorescence of rhodamine 6G was used for calibration because it did not depend on the amount of glucose. Azmi et al. [88] applied the same principle to detecting uric acid. They used mercaptopropionic acid-caped CdS NCs conjugated with uricase and HRP and embedded in a hydrogel based on aminopropyl trimethoxysilane, 3-glycidoxypropyl trimethoxysilane, and ethanol. In the presence of uricase, uric acid was oxidized to form, among other products, H_2_O_2_, which quenched the NC fluorescence. A high specificity of the detection of biological compounds that themselves have no optical activity is an undoubted advantage of this method; however, it is more difficult to implement and can be used only if there is a specific oxidase for the analyte. Fluorescent hydrogel biosensors employing different detecting mechanisms are being intensely developed by a number of research groups (Table 1).

**Figure 5:**
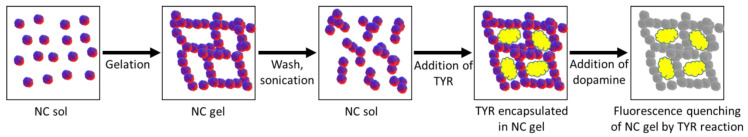
Scheme of the encapsulation of enzymes in a NC gel using sol–gel switching process of MSA-capped CdTe NCs. The Scheme is adapted from Ref. [89].

The miniature size of the biosensors imposes significant limitations on the area available for immobilization of sensor or affinity labels recognizing specific analytes. Switching from the 2D detection model to the 3D one by using 3D polymer matrices not only increases the capacity for binding the sensor and affinity labels, but also preserves the structure of the molecules, which could denature upon immobilization on a flat surface [91]. This prevents the deterioration of the properties necessary for sensing, such as specificity and sensitivity [92]. All of this makes it possible to increase the detection sensitivity of microfluidic biosensors compared to their full-size counterparts. When the signal or recognition labels are immobilized in the 3D structure of the hydrogel, their orientation becomes irrelevant, because the analyte passes on all sides of them in any case, which increases the probability of the detection. For example, Gao et al. [93] have shown that the efficiency of DNA hybridization on a surface is 20–40 times lower than in a solution. When antibodies are immobilized on a flat surface, they can also lose specificity and affinity for their antigens, which is confirmed by numerous examples reviewed by Welch et al. [94]. Feng et al. [95] have shown that 3D arrangement of antibodies even on a flat surface can increase the detection sensitivity by a factor of 64. In addition, the hydrogel structure itself can perform a sensory function. For example, the selectivity for the analyte size or selectivity of the analyte–hydrogel interaction can be increased, or nonspecific binding of the analyte decreased, by varying the pore size of the 3D matrix or by selecting materials with different physical and chemical properties for its formation. Yuan et al. [89] developed NC hydrogels with encapsulated tyrosinase (TYR) for biosensing of dopamine (Figure 5). The use of a neutral phosphate-buffered saline to dissolve the precipitated CdTe NCs capped with mercaptosuccinic acid (MSA) sufficiently shortened the NC gelation time (to several days), and the sol–gel transition was also observed in the as-prepared NC gels. The resulting gels had pores ranging from 10 to 50 nm in diameter, and the enzyme could be encapsulated in the mesopores of the gel network during the gelation. The encapsulation of TYR was confirmed by atomic force microscopy and the test reaction of tyrosinase-catalyzed oxidation of catechol. The enzymatic activity of TYR in the hydrogel was preserved even after immersion in a potassium buffer solution for at least one week. Tyrosinase catalyzes the oxidation of dopamine to dopamine-o-quinone, which quenches NC fluorescence. Addition of dopamine quenched the NC fluorescence in the tyrosinase-embedded hydrogel, but it had no obvious effect on the pure NC hydrogel in the absence of tyrosinase. The detection limit for dopamine was found to be 5.0 × 10^−8^ mol L^−1^, the practicable range of detection being from 5.0 × 10^−5^ to 1.0 × 10^−3^ mol L^−1^. This dopamine detection limit was four times lower than that determined for the CdTe NC sol [96] and comparable with those of electrochemical assays [97].

Thus, the use of a 3D matrix for immobilizing the labels and detecting the analytes could significantly increase the detection sensitivity due to both increased numbers of fluorescent and affinity labels and an increased efficiency of binding the analyte by the affinity labels.

There are two main types of emerging biosensors based on NC-doped hydrogels. The first one includes microfluidic devices where the sample is passed through a microchannel filled with a hydrogel. Jang et al. [98] developed a microfluidic biosensor of glucose and alcohol. The biosensor consisted of three components hierarchically integrated into a microfluidic device: NCs conjugated with glucose oxidase (GOX) or alcohol oxidase (AOX), hydrogel microstructures, and microchannels. The hydrogel-entrapped GOX or AOX catalyzed the oxidation of glucose and alcohol, respectively, to produce H_2_O_2_, which subsequently quenched the fluorescence of the conjugated NCs. The hydrogels were fabricated from poly(ethylene glycol) diacrylate (PEG-DA) via light-induced polymerization. PEG-DA was dissolved in phosphate-buffered saline and mixed with NC–oxidase conjugates and 2-hydroxy-2-methylpropiophenone serving as a photoinitiator. After the microchannels were filled with precursor solutions, a photomask was placed on the other side of each glass slide to obtain the desired hydrogel pattern in the microfluidic channels. After UV exposure and hydrogel polymerization, the microfluidic channels were washed to obtain immobilized hydrogel microstructures entrapping the NC–oxidase conjugates. The analyte (glucose or ethanol) was continuously supplied into the microchannels at a rate of 1.0 L/min. The detection limits of this biosensor were found to be 50 μM for glucose and 70 μM for ethyl alcohol. This sensor can be adapted for multiplexed detection by immobilizing different NC–oxidase conjugates in different microfluidic channels. The most commonly used polymeric microfluidic chips are made of thermoset plastics, such as phenol formaldehyde and epoxy resins, or thermoplastic materials, such as polystyrene, polyetherketones, polyvinyl chloride, polycarbonate, and polydimethylsiloxane. Polymeric microfluidic chips are resistant to chemical agents, easy to chemically modify, and cheap to manufacture. Inorganic fluorescent gels can be immobilized inside microfluidic systems by two methods. The first one is based on gelation inside the assembled microfluidic chip containing a cavity into which a colloidal solution of NCs and affinity molecules is fed, and then gelation is initiated by either adding a gel-forming chemical agent or irradiating the chip with light [98]. The second method is to apply the gel onto the surface of the plate immediately after its fabrication, before closing the microfluidic channels, and then to complete the assembly of the microfluidic system. In the latter case, the colloidal solution is applied using a method similar to inkjet printing [99].

Another promising type of hydrogel-based biosensor is a hydrogel optical fiber. For example, Zhou et al. [89] developed a ratiometric fluorescence sensor for the detection of Fe^3+^ ions based on quantum-dot-doped hydrogel optical fibers. They used gelation inside a silicone tube mold, as in the microfluidic devices mentioned above. There were two types of CdTe NCs with different emission bands: NCs that were coated with thioglycolic acid, emitted green fluorescence, and were insensitive to metal ions, which therefore served as a reference (gNCs), and Fe^3+^-specific NCs coated with N-acetyl-L-cysteine that emitted red fluorescence and whose fluorescence was highly selectively quenched by Fe^3+^ ions (rNCs). NCs of both types were prepared by mixing with polyethylene glycol diacrylate and 2-hydroxy-2-methyl-propiophenone and were then injected into the silicone tube mold by means of a syringe to form the fiber core. For obtaining light coupling, a multimode silica fiber was pigtailed to the hydrogel fiber by inserting a short section of the silica fiber end into the precursor and aligning it to the center of the tube mold. A UV lamp was used for photo-crosslinking of the precursors. After 5 min of irradiation, the polymerized hydrogel fiber pigtailed with the silica optical fiber was pulled out of the tube mold. The dip-coating technique was used to form the cover, for which purpose the fiber core was first dipped into a sodium alginate solution and then immersed in a CaCl_2_ solution to induce crosslinking of alginate chains. By ratiometric detection of the intensities of fluorescence of the two NC types, quantitative and selective detection of Fe^3+^ ions was achieved in a linear range of 0–3.5 μM, with a detection limit of 14 nM. A similar approach was used by Li et al. [100] for engineering a difunctional hydrogel optical fiber fluorescence sensor for continuous and simultaneous monitoring of the glucose level and pH.

## 5. Conclusions

Biosensors based on composite inorganic fluorescent hydrogels offer a number of advantages over the state-of-the-art microfluidic and immunochromatographic tests, which are often used in PoC diagnosis. In contrast to immunochromatographic tests, biosensors based on composite inorganic fluorescent hydrogels have a 3D structure, which allows immobilizing a larger number of capture molecules, preserving their structure, and orienting all analyte-binding sites towards the solution to ensure the maximal avidity of capture structure. In addition, the use of a fluorescent signal increases the sensitivity of analyte detection compared to chromogenic staining methods. With the change in the fluorescence signal level being reversible, biosensors based on inorganic fluorescent hydrogels can be operated in the flow mode for continuous monitoring or for sequential examination of several samples. Microfluidic systems have several advantages over their full-size counterparts, including a high throughput, a low manufacturing cost, the use of small volumes of the sample, and a short time of analysis. Another promising type of biosensor includes the devices based on NC-doped hydrogel optical fibers, with a polymerized hydrogel fiber pigtailed with a silica optical fiber to ensure both excitation and detection of the fluorescent signal. This design of hydrogel-based biosensors makes it possible to study flowing samples, or static samples when the biosensor is immersed in a solution. To date, hydrogel-based biosensors can already be used to detect metal ions, enzymes, reactive oxygen species, some biologically active molecules, and markers of diseases and pathological conditions, as well as hazardous contaminants in food and the environment. There are grounds to believe that further developments in this field will be aimed at increasing the specificity of detection and extending the list of detectable analytes, which will increase the demand for biosensors based on composite inorganic gels in the PoC diagnosis segment.

## Figures and Tables

**Table 1:** Biosensors based on fluorescent hydrogels.

Target	Fluorescent Tag	Gel Material	Detection Limit	Detection Principle	Ref.
Hexavalent chromium (Cr(VI))	CDs	Lignin and cellulose nanofibers	11.2 mg/L	Fluorescence quenching by chromium ions	[78]
Hexavalent chromium (Cr(VI))	CDs	Polyvinylpyrrolidone	1.2 µM	Fluorescence quenching by chromium ions	[76]
Silver ion (Ag^+^) and other metal ions	CDs	BMIM-BF_4_	0.55 µg/mL for Ag^+^	Fluorescence quenching by metal ions	[68]
Iron ion (Fe^3+^)	CDs	Crosslinked microcrystalline cellulose	65 nM	Fluorescence quenching by iron ions	[75]
Nitrite (NO_2_^−^)	MPD-modified CDs	Agarose	0.018 μM	Fluorescence quenching by NO_2_^−^	[70]
Tetracycline	CDs	Alginate	2 μM	Fluorescence quenching by tetracycline	[79]
Glucose	CDs	Acrylic acid and diacrylated PEG	0.04 μM	Fluorescence quenching mediated by glucose oxidase and HRP	[86]
*Bacillus* and *Staphylococcus* strains	CDs	DTG	10^5^ cells/mL for *B. cereus*	Fluorescence quenching caused by aggregation of CDs	[71]
Progesterone	CdSe/CdS/ZnS NCs	PEG	55 nM	Fluorescence quenching by progesterone-mediated FRET disruption	[85]
Dopamine	MSA-capped CdTe NCs	Gelated MSA-capped CdTe NCc	50 nmol/ L	Fluorescence quenching mediated by tyrosinase	[89]
Uric acid	MPA-caped CdS NCs	APTMS, GPTMS, and ethanol	50 µM	Fluorescence quenching mediated by uricase and HRP	[88]
Iron ion (Fe^3+^)	CdTe NCs	PEG-DA and HMP	14 nM	Fluorescence quenching by iron ions	[90]
Polyaromatic compounds (R101, 2-MN, and NA)	GQDs	AETA and MBA	-	Fluorescence quenching by polyaromatic compounds	[69]
Laccase	Sulfur- and nitrogen-codoped GQDs	Nanocellulose	0.048 U/mL	Fluorescence quenching mediated by laccase	[80]

CDs, carbon dots; BMIM-BF_4_, 1-butyl-3-methylimidazolium tetrafluoroborate; MPD, m-phenylenediamine; PEG, poly(ethylene glycol); HRP, horseradish peroxidase; DTG, 6-O-(O-O′-dilauroyltartaryl)-D-glucose; NCs, nanocrystals; FRET, Förster resonance energy transfer; MSA, mercaptosuccinic acid; MPA, mercaptopropionic acid; APTMS, aminopropyl trimethoxysilane, GPTMS, 3-glycidoxypropyl trimethoxysilane; PEG-DA, polyethyleneglycol diacrylate; HMP, 2-hydroxy-2-methyl-propiophenone; R101, rhodamine 101; 2-MN, 2-methoxynaphthalene; NA, sodium 1-naphthalenesulfonate; GQDs, graphene quantum dots; AETA, [2-(acryloyloxy)ethyl]trimethyl-ammonium chloride; MBA, N,N′-methylenebisacrylamide.

## Data Availability

Not applicable.
